# Outcomes of Robot Assisted Trans Abdominal Retromuscular Umbilical Prosthesis (rTARUP): A Dutch Multicenter Study

**DOI:** 10.3389/jaws.2025.15479

**Published:** 2025-12-22

**Authors:** Julotte E. Baart, Didi A. M. Sloothaak, Maarten P. Simons, Alexander L. Bloemendaal

**Affiliations:** 1 Department of Surgery, Reinier de Graaf Hospital, Delft, Netherlands; 2 Department of Surgery, Spaarne Gasthuis Hoofddorp, Hoofddorp, Netherlands; 3 Department of Surgery, OLVG, Amsterdam, Netherlands

**Keywords:** TARM, rTARUP, abdominal wall hernia, robotic abdominal wall repair, ventral hernia repair

## Abstract

**Aim:**

Robotic assisted Trans Abdominal Retromuscular Umbilical Prosthesis (rTARUP) is being increasingly performed to treat Ventral Hernia. Prospective (long-term) studies are scarce. In this study we investigated 125 patients with short and long-term follow-up, regarding surgical and hernia-related quality-of-life outcomes.

**Material and Methods:**

Patients undergoing rTARUP were selected from consecutive prospectively collected databases from two teaching hospitals from the Netherlands. The patients were operated by experienced surgeons in a standardized manner. Patient characteristics and short-term outcomes after surgery were retrospectively reviewed and follow up data about hernia recurrence and quality of life was prospectively collected.

**Results:**

125 rTARUP patients were included for analysis. The most common hernia type was primary (n = 61), followed by recurrent (n = 37) and incisional (n = 27). The median surgical time was 90 min. 44/61 (72%) patients with a primary ventral hernia were treated as daycase. The 30-day complication rate was 7.2%. Median follow up was 24 months after which a large majority of patients (84%) reported to feel better or much better. There were 3 reoperations for recurrences (2.4%).

**Conclusion:**

rTARUP is a safe and effective procedure that can be performed in a timely manner, with short hospital stay and a low complication rate. Further research is needed into economical costs and benefits to fully justify the use of robotic surgery for these indications.

## Introduction

Ventral abdominal wall hernia has an estimated prevalence of 5% in the general population [1] and is increasingly prevalent due to an aging and increasingly obese society, receiving more abdominal surgery [2]. The Rives Stoppa procedure is a proven efficacious treatment for ventral abdominal wall hernia and the retrorectus mesh placement may be considered most favorable [3, 4]. In 2022, a systemic review showed superior outcomes compared to other techniques. However, surgical site infection was less prevalent in intraperitoneal onlay mesh (IPOM) repair, clearly benefitting from the minimally invasive approach [3]. The IPOM technique was a first attempt at minimally invasive hernia repair, seeking benefits of less extensive dissection in the subcutaneous tissue, shorter hospital stays, and fewer surgical site infections. However, complications such as recurrences, bulging, formation of intraperitoneal adhesions, fistulas, and bowel damage are known complications of IPOM repair [5, 6].

Combining the benefits of a minimally invasive (laparoscopic) approach and retromuscular mesh position, Schroeder et al. described a laparoscopic transabdominal technique: the Trans Abdominal Retrorectus Umbilical Prosthesis (TARUP). Authors concluded that the new approach was a safe and effective alternative to open Rives Stoppa, but also a technically demanding procedure [4]. In 2018, Muysoms et al. reported on the robotically assisted TARUP procedure (rTARUP). This adaptation of Schroeder’s laparoscopic approach showed similar complication rates and shorter operative times [7].

Since 2018, only a handful of reports on rTARUP (or now also known as rTARM, Trans Abdominal Retromuscular Mesh) has been published, with limited number of patients and limited follow up. Garza et al. reported on a 101 patient series with 3 years follow-up showing low recurrence rates of 3% and improvement in quality of life according to the EuraHS Quality-of-Life scale [8]. Vierstraete et al. reported a similar recurrence rate of 3.7% at a median follow up of 4.5 years of 162 patients, with a complication rate of 6.2% [9].

We believe rTARUP/rTARM may be considered an important improvement in the treatment of ventral abdominal wall hernias. This study reports on technical aspects, short and long-term outcomes and quality of life of rTARUP. In addition, we comment on lessons learned since the implementation of rTARUP in two teaching hospitals in the Netherlands.

## Methods

### Setting

The study was conducted in the Reinier de Graaf Gasthuis (RdGG) and OLVG hospital in Amsterdam. Both hospitals are district teaching hospitals with expertise in robotic surgery with the Intuitive Da Vinci Surgical platform. Patients included for this study were either operated by MS (OLVG) or BB (RdGG).

### Patient Selection

Both hospitals follow the EHS guidelines that recommend very small umbilical hernia to be treated either by primary suture or open preperitoneal mesh [10]. The decision to perform rTARUP was based on the surgeon’s discretion. In general, patients were considered for rTARUP (instead of open or laparoscopic techniques) if there were multiple hernias, or if rectus abdominis diastasis was present in a primary ventral hernia. Both elective and emergency rTARUP procedures were eligible for inclusion in RdGG, in OLVG only elective procedures were included. Patients undergoing rTARUP solely for rectus abdominis diastasis without concomitant ventral hernia were excluded from analysis, as well as patients receiving a combined operation consisting of rTARUP and another operation (i.e., rTAPP).

### Data Collection

Data from patients undergoing rTARUP were prospectively collected in databases in RdGG (February 2020 to January 2024) and OLVG (September 2019 and August 2023). Missing data were collected through patient-file review in accordance with the Medical Ethics Committee of both institutions. The following data were registered: age at time of operation, gender, comorbidities, previous abdominal surgery including history of recurrent hernias, American Society of Anesthesiologists (ASA) classification of physical status, BMI, smoking, and immunosuppressant use. Hernia characteristics were described as: type of hernia (primary, incisional or recurrent), hernia multiplicity and size, concurrent diastasis classification according to Reinpold et al. [11], hernia classification according to the European Hernia Society descriptive classification (Supplementary Appendix S1) [12] and hernia stage according to HPW-classification for staging (Supplementary Appendix S2) [13]. Surgical outcomes included intra-operative complications, surgical time, hospital stay and 30-day complications according to Clavien Dindo [14].

Follow up data was collected after patient permission was obtained. In October and November of 2024, all patients were contacted by telephone for additional follow-up via the EuraHS Quality of Life-questionnaire (EuraHS-QoL) [15]. In OLVG patients, the questionnaire was discussed via telephone in Dutch or English. In RdGG patients, a digitalized, Dutch version was sent out to participants. Patients who did not speak English or Dutch were excluded from the EuraHS-QoL data-collection. If suspicion of a recurrence was present during telephonic follow up, patients were scheduled for an in-office appointment. Recurrences were confirmed by physical examination and/or if indicated by ultrasound or CT-scan.

### Surgical Technique and Peri-Operative Care

Surgeries were performed by two experienced robotic surgeons who performed rTARUP according to the standardized technique described by Muysoms [7], using a ProGrip [Medtronic, Fridley, USA] mesh as reinforcement. V-loc 0 slowly (180 days) absorbable [Medtronic, Fridley USA] was used for closure of the anterior fascia, and a V-loc 3/0 slowly (180 days) absorbable [Medtronic, Fridley USA] for the posterior fascia. Rectus diastasis was plicated in almost all patients. Patients at RdGG were scheduled for at least one postoperative night, as they received intra-thecal morphine and a urinary catheter. In OLVG, most patients were scheduled as outpatient procedures. Post-operative care in both centers consisted of oral non-opioid analgesics, wearing of an abdominal binder for the first 24 h post-operatively and during mobilization for at least the first 4 weeks, and a ban on lifting and heavy exercising for the first 6 weeks.

### Statistical Analysis

Data were analyzed using IBM SPSS Software, PASW Statistics version 29.0.2.0. Categorical variables were analyzed using Pearson Chi^2^, Fisher’s exact- and Fisher-Freeman-Halton Exact test. Continuous data were analyzed for normalcy with Shapiro-Wilk and Q-Q plots to decide on (non)parametric tests: unpaired t-test, one-way ANOVA, Mann Whitney U test and Kruskale Wallis non-parametric analysis. Mean values were presented with standard deviation (SD) and median values with inter-quartile range (IQR). Baseline characteristics and surgical outcomes were compared between OLVG and RdGG and compared within hernia type (primary, incisional, and recurrent). P-values <0.05 (two-sided) were considered statistically significant.

### Subgroup Analysis and Classification

For further analysis, we decided to group the ventral hernias in three groups: primary, incisional and recurrent hernias. Recurrent hernias were defined as primary or incisional hernias which had already been corrected (at least) once. Incisional hernias that had been corrected before, also fell into the recurrent group. Recurrent hernia is considered a risk factor for a new recurrence. This can be due by either underlying dysfunction in the wound healing process, such as abnormal collagen type I/type III ratio or other genetic connective tissue disorders, or due to repeated hernia repairs which cumulatively decrease the quality of the abdominal wall tissue, resulting in increasingly vulnerable reconstructions [16]. It has been shown that the risk of recurrence increases with every repair [16, 17]. This is why we decided to analyse this group of patients separately, even though this deviates from the EHS-classification [12] which defines a recurrence as an incisional hernia irrespective of the number of recurrent operations. Even though our classification system is not standard, other authors have also proposed to include recurrence status into classification systems or as a separate class itself [16, 18–21].

## Results

### Comparison of Baseline Characteristics of Patients Between Centers

Table 1 shows a comparison of baseline characteristics between the two including centers. Patients from RdGG presented significantly more often with a history of previous abdominal surgery compared to patients from OLVG. At OLVG, most patients had a primary hernia, while recurrent hernias were more common in patients from RdGG. Almost all patients from OLVG were diagnosed with rectus diastasis as opposed to a large majority with concomitant rectus diastasis at RdGG. At RdGG the number of previous repairs in recurrent hernias was significantly higher than at OLVG.

**TABLE 1:** Baseline characteristics of included patients (n = 125).

Baseline characteristics	OLVG (n = 69)	RDGG (n = 56)	P-value
Female (%)	42 (60.9)	31 (55.4)	0.586^a^
Age in years^b^ (SD)	51.32 (12.866)	53.80 (13.212)	0.293^c^
BMI^d^ (IQR)	26.85 (23.94–30.44)	28.70 (24.32–32.29)	0.141^e^
Current smoker (%)	15 (28.3)	9 (16.4)	0.168^a^
Immuno-suppressant user (%)	4 (5.8)	2 (3.6)	0.690^a^
Previous abdominal surgery (%)	40 (58.0)	46 (82.1)	0.004^a^
ASA classification (%)			0.726^f^
I	25 (36.2)	19 (33.9)	
II	33 (47.8)	25 (44.6)	
III	11 (15.9)	12 (21.4)	
Multiple hernias (%)	34 (49.3)	23 (41.1)	0.360^g^
Primary, incisional or recurrent (%)			<0.001^f^
Primary	45 (65.2)	16 (28.6)	
Incisional	12 (17.4)	15 (26.8)	
Recurrent	12 (14.4)	25 (44.6)	
EHS width (%)			<0.001^f^
W1 (primary <2 cm, recurrent <4 cm)	40 (74.1)	23 (41.8)	
W2 (primary 2–4 cm, recurrent 4–10 cm)	11 (20.4)	32 (58.2)	
W3 (primary >4 cm, recurrent >10 cm)	3 (5.6)	0 (0)	
HPW stage			0.465^f^
1	44 (64.7)	33 (58.9)	
2	23 (33.8)	20 (35.7)	
3	1 (1.5)	3 (5.4)	
Width in cm^d^ (IQR)	1.68 (0.98–3.33)	3.00 (2.00–5.00)	<0.001^e^
Length in cm^d^ (IQR)	3.50 (1.00–6.00)	4.00 (2.63–7.50)	0.048^e^
Rectus diastasis present (%)	68 (98.6)	44 (78.6)	<0.001^a^
Diastasis width in cm^d^ (IQR)	4.2 (3.2–5.5)	5.0 (3.5–6.0)	0.313^e^
Diastasis classification (%)			0.784^g^
D1 (2–3 cm)	11 (16.2)	8 (18.2)	
D2 (3–5 cm)	36 (52.9)	20 (45.5)	
D3 (>5 cm)	21 (30.9)	16 (36.4)	
Number of previous hernia repairs			0.003^f^
0	57 (82.6)	31 (55.4)	
1	10 (14.5)	18 (32.1)	
2–4	2 (2.9)	7 (12.5)	

^a^ Fisher’s Exact Test (2-sided).

^b^ normal distribution according to Shapiro-Wilk

^c^ Independent t-test (2-sided).

^d^ not-normal distribution according to Shapiro-Wilk.

^e^ Mann-Whitney Test.

^f^ Fisher-Freeman-Halton Exact test: (2-sided).

^g^ Pearson-Chi Square Test (2-sided).

^h^ One-way ANOVA.

^i^ Kruskal-Wallis Test: (Asymp).

SD, standard deviation; IQR, inter-quartile range; BMI, body mass index; ASA, american society of anesthesiologists; EHS, width, hernia width classified according to the European Hernia Society; HPW, stage, hernia staging according to the Hernia, Patient, Width classification.

### Subgroup Analysis

Table 2 shows a comparison of baseline characteristics between the subgroups of primary, incisional or recurrent hernias. The incisional hernia group was significantly older. Most patients with a primary hernia were either ASA I or II, while most patients with an incisional hernia were either II or III. In all patients with recurrent hernias, 28 patients (75.7%) had undergone one previous hernia repair, nine patients had undergone more than one repair already (varying between two and four repairs). In the recurrent hernias, 18 patients (48.6%) already had a mesh present in the abdominal wall, and two patients (5.4%) had two meshes present. One patient with a primary epigastric hernia, already had an umbilical hernia mesh in place.

**TABLE 2:** Comparison of baseline characteristics between subgroups.

Characteristics	Primary (n = 61)	Incisional (n = 27)	Recurrent (n = 37)	P-value
Female	37 (60.7)	13 (48.1)	23 (62.2)	0.495^a^
Age in years^b^ (SD)	47.65 (11.912)	59.81 (12.830)	54.84 (11.985)	<0.001^c^
BMI^d^ (IQR)	26.85 (23.94–31.18)	27.34 (24.38–31.62)	27.99 (24.05–31.74)	0.699^e^
Smoker (%)	8 (16.3)	8 (33.3)	8 (22.9)	0.245^a^
Immuno-suppressant user (%)	4 (6.6)	1 (3.7)	1 (2.7)	0.862^a^
Previous abdominal surgery (%)	22 (36.1)	100%	100%	<0.001^a^
ASA (%)				0.003^a^
I	28 (45.9)	2 (7.4)	14 (37.8)	
II	26 (42.6)	18 (66.7)	14 (37.8)	
III	7 (11.5)	7 (25.9)	9 (24.3)	
Multiple hernias (%)	24 (39.3)	17 (63.0)	16 (43.2)	0.122^f^
EHS width (%)				0.347^a^
W1	27 (54.0)	17 (70.8)	19 (54.3)	
W2	20 (40.0)	7 (29.2)	16 (45.7)	
W3	3 (6.0)	0	0	
EHS zone (%)				0.001^a^
M1 subxyphoidal	0	1 (3.8)	0	
M2 epigastric	17 (28.8)	6 (23.1)	16 (42.3)	
M3 umbilical	42 (71.2)	13 (50.0)	18 (48.6)	
M4 infraumbilical	0	1 (3.8)	0	
Combination		5 (19.2)	3 (8.1)	
HPW stage (%)				0.119^a^
1	37 (61.7)	15 (55.6)	25 (67.6)	
2	23 (38.3)	9 (33.3)	11 (29.7)	
3	0	3 (11.1)	1 (2.7)	
Width in cm^d^ (IQR)	1.55 (1.00–3.00)	3.50 (2.70–5.75)	3.0 (11.95–5.00)	<0.001^e^
Length in cm^d^ (IQR)	3.0 (1.00–4.80)	7.45 (3.85–10.75)	3.40 (2.00–5.93)	0.014^e^
Rectus diastasis present (%)	58 (95.1)	24 (88.9)	30 (81.1)	0.069^a^
Diastasis width in cm^d^ (IQR)	4.5 (3.45–5.85)	4.25 (3.15–5.76)	4.00 (3.00–6.00)	0.663^e^
Diastasis width classification (%)				0.647^a^
D1 (2–3 cm)	8 (13.8)	6 (25.0)	6 (16.7)	
D2 (3–5 cm)	28 (48.3)	11 (45.8)	17 (57.7)	
D3 (>5 cm)	22 (37.9)	7 (29.2)	8 (26.7)	

^a^ Fisher-Freeman-Halton Exact test: (2-sided).

^b^ normal distribution according to Shapiro-Wilk

^c^ One-way ANOVA.

^d^ not-normal distribution according to Shapiro-Wilk.

^e^ Kruskal-Wallis Test: (Asymp).

^f^ Pearson-Chi Square Test (2-sided).

SD, standard deviation; IQR, inter-quartile range; ASA, american society of anesthesiologists; EHS, european hernia society; HPW, hernia patient wound; cm, centimeter.

### Surgical Outcomes

Table 3 summarizes operative outcomes. ProGrip [Medtronic Fridley USA) mesh was used in all but one cases. In one case a Dynamesh [DynaMesh, Aachen, Germany] was used.

**TABLE 3:** Operative outcomes between centers.

Operative outcomes	OLVG	RDGG	Total	P-value
Operating time in minutes^a^ (IQR)	146 (120–180)	121 (99–140)	134 (115–160)	<0.001^b^
Skin to skin time in minutes^a^ (IQR)	110 (90–133)	82 (65–100)	90 (78–123)	<0.001^b^
Intra-operative complications (%)	1 (1.4)	0	1 (0.8)	1.00^c^
Mesh width in cm^a^ (IQR)	12 (11–15)	14 (12–15)	13 (12–15)	0.007^b^
Mesh length in cm^a^ (IQR)	20 (15–20)	18 (15–22)	19 (15–22)	0.410^b^
Length of stay (%)				<0.001^d^
Day care	33 (47.8)	0	33 (26.4)	
1 post-operative night	30 (43.5)	4 (7.1)	34 (27.2)	
2 post-operative nights	5 (7.2)	34 (60.7)	39 (31.2)	
3 post-operative nights	1 (1.4)	14 (25.0)	15 (12.0)	
4 post-operative nights	0	2 (3.6)	2 (1.6)	
5 post-operative nights	0	2 (3.6)	2 (1.6)	
30 days complications (%)	5 (7.2)	4 (7.1)	9 (7.2)	1.00^c^
Clavien dindo score				0.148^d^
0	63 (91.3)	50 (89.3)	113 (90.4)	
I	4 (5.8)	3 (5.4)	7 (5.6)	
II	2 (2.9)	0	2 (1.6)	
III	0	3 (5.4)	3 (2.4)	
Reoperation for hernia recurrence (%)	1 (1.4)	2 (3.6)	3 (2.4)	0.587^c^
Diastasis recurrence (%)	2 (2.9)	0	2 (1.6)	0.501^c^
Reoperation (%)				0.501^d^
1	2 (2.9)	3 (5.4)	5 (4.0)	
2	0	1 (1.8)	1 (0.8)	
Length of follow up in months^a^ (IQR)	27 (18–38)	22 (15.5–35.5)	24 (17–36)	0.172^b^

^a^ not-normal distribution according to Shapiro-Wilk.

^b^ Mann-Whitney Test.

^c^ Fisher’s Exact Test (2-sided).

^d^ Fisher-Freeman-Halton Exact test: (2-sided).

IQR, inter-quartile range; cm, centimeter.

In one patient the anterior fascia suture snapped during the emergence from anesthesia. The patient was reintubated to perform open surgery to re-close the fascia. There were four minor 30-day complications without need for intervention: two patients with seroma, one with hematoma, and one patient who had temporary urine retention. Other minor complications include a patient with gastroparesis, which resolved shortly upon the use of a nasogastric tube. Another patient experienced ACNES-like pain at the trocar site and received a lidocaine injection and is currently pain free.

Three patients experienced major complications. One patient, who was a smoker and morbidly obese, was treated for a fourth hernia recurrence and developed an infected seroma, ultimately necessitating surgical debridement, vacuum assisted wound therapy and IV antibiotics. Another patient experienced a superficial wound infection after a procedure in which the infected mesh from a prior procedure was removed and was treated successfully as described above. One patient developed a paralytic ileus and fever 2 days post-operatively, which was treated with antibiotics and a nasogastric tube. Because of stagnating recovery, a CT-scan was performed showing free air and fluid. Explorative laparotomy showed a perforated small bowel due to strangulation/herniation between the sutures in the posterior fascia. The mesh was removed due to bowel content contamination. Post-operatively the patient developed a pneumonia and an abscess which was drained and treated with intravenous antibiotics and vacuum assisted closure. Months after full recovery and having developed a recurrent hernia, patient underwent an open reconstruction with an intraperitoneally placed biological mesh [Ovitex; TelaBio, Malvern USA], after which the patient recovered well.

During follow-up, hernia recurrence was found in three patients and diastasis recti was found in two patients. Two hernia recurrences occurred cranially from a mesh where the rectus diastasis was not fully approximated. All patients with clinically proven recurrence underwent re-operation. Because not all patients received physical examination at 2 years follow up, we labeled these recurrences as ‘reoperation rate for recurrence’.

There were 6 reoperations in total. One small recurrent hernia was localized cranially to the mesh and was closed primarily under local anesthesia. Another patient received IPOM repair for a recurrence. The patient with the bowel perforation was operated on twice: once for the bowel perforation and once for the following recurrence. Two reoperations were due to wound complications which required debridement and application of vacuum assisted closure. Lastly, one patient underwent surgery for the excision of a symptomatic sub xiphoid calcification, which turned out to be post traumatic heterotopic ossification as a reaction to previous hernia repair.

### Outcome Differences Between Centers

**FIGURE 1:**
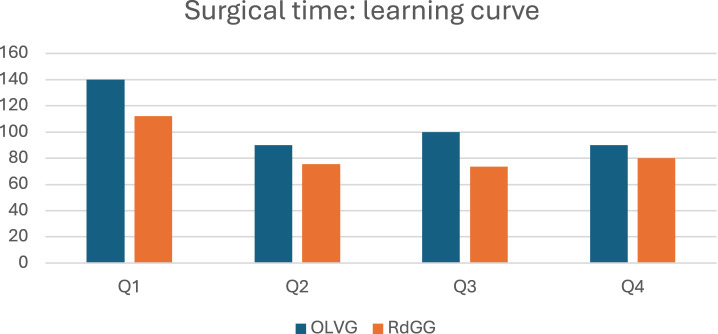
Learning curve in median surgical time in minutes per consecutive quartile. Quartiles (Q) consist of 17 patients at OLVG and of 14 patients at RdGG.

**FIGURE 2:**
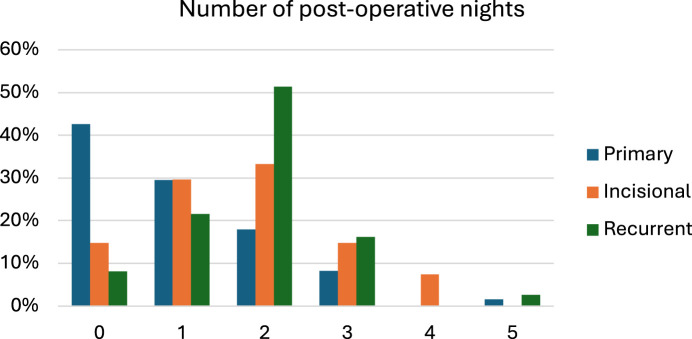
Length of stay (0: day care).

Table 3 shows surgical outcomes specified by center. Figure 1 represents the learning curves in surgical time. All patients operated in day treatment were operated in OLVG. Operating times were significantly lower in RdGG. The learning curves in surgical time are shown in Figure 2.

### Comparison of Outcomes Between Subgroups

Surgical outcomes of subgroups are summarized in Table 4. Length of stay differed significantly between groups (P < 0.001) and is described in Figure 2.

**TABLE 4:** Surgical outcomes of subgroups.

Operative outcomes	Primary	Incisional	Recurrent	P-value
Operating time in minutes^a^ (IQR)	130 (110–160)	140 (120–160)	134 (119–173)	0.460^b^
Skin to skin time in minutes^a^ (IQR)	90 (75–120)	110 (79–120)	94 (80–134)	0.415^b^
Intra-operative complications (%)	1 (1.6)	0	0	1.000^c^
Mesh width in CM^a^ (IQR)	12 (11–15)	13 (12–15)	13 (12–15)	0.155^b^
Mesh length in CM^a^ (IQR)	20 (15–22)	20 (15–24)	16 (14.5–21)	0.131^b^
30 days complications (%)	1 (1.6)	2 (7.4)	6 (16.2)	0.071^c^
Clavien dindo score				0.104^c^
0	58 (95.1)	24 (88.9)	31 (83.8)	
I	2 (3.3)	3 (11.1)	2 (5.4)	
II	1 (1.6)	0	1 (2.7)	
III	0	0	3 (8.1)	
Reoperation for hernia recurrence (%)	1 (1.6)	0	2 (5.4)	0.440^c^
Diastasis recurrence (%)	2 (3.3)	0	0	0.709^c^
Reoperation (%)				0.242^c^
1	2 (3.3)	0	3 (8.1)	
2	0	0	1 (2.7)	
Length of follow up in months^a^ (IQR)	23 (17–35)	28 (16–47)	24 (17–41)	0.911^b^

^a^ not-normal distribution according to Shapiro-Wilk.

^b^ Kruskal-Wallis Test: (Asymp).

^c^ Fisher-Freeman-Halton Exact test: (2-sided).

IQR, inter-quartile range.

### Quality of Life

Quality of life outcomes are shown in Table 5. The response rate for the quality-of-life questionnaire was 77.6% (n = 97). During telephonic follow up, six patients mentioned still feeling pain at the operation site. Two patients experienced scar tissue pain. In the other patients, investigations have ruled out recurrence, mesh infection or other complications. Because the cause of the pain remains unclear, two of them have been diagnosed with Chronic Postoperative Pain and were referred to a specialized pain-clinic.

**TABLE 5:** Quality of life after surgery.

Quality-of-life outcomes	Primary	Incisional	Recurrent	P-value
QoL total score (/90)^a^ (IQR)	9 (2.5–23.5)	6.66 (0–22.5)	10 (3.25–29.0)	0.637^b^
QoL domain pain (/30)^a^ (IQR)	0 (0–4)	0 (0–7)	0.5 (0–5)	0.511^b^
QoL domain restrictions (/40)^a^ (IQR)	0 (0–8)	1 (0–6.7)	1 (0–13)	0.615^b^
QoL domain cosmesis (/20)^a^ (IQR)	4 (0–10)	4 (0–10)	5 (0.5–10)	0.896^b^
Patient-reported pain (%)	4 (6.6)	1 (3.7)	1 (2.7)	0.862^c^
Worse or better off (%)				0.930^c^
Much worse	1 (2.2)	0	1 (5.3)	
Worse	1 (2.2)	0	0	
The same	5 (11.1)	3 (18.8)	2 (10.5)	
Better	19 (42.2)	6 (37.5)	6 (31.60	
Much better	19 (42.2)	7 (43.8)	10 (52.6)	

^a^ not-normal distribution according to Shapiro-Wilk.

^b^ Kruskal-Wallis Test: (Asymp).

^c^ Fisher-Freeman-Halton Exact test: (2-sided).

QoL, Quality of Life score according to the EuraHS-Quality of Life questionnaire; IQR, inter-quartile range.

An overview of quality-of-life outcomes specified by center, is available in Supplementary Appendix S4. Overall quality-of-life scores were comparable between centers. The total domain scores for pain and restrictions differed slightly. Median pain score at RdGG was 2/30 (IQR 0–9) and 0/30 (IQR 0–2) at OLVG. Median restriction score was 4 (IQR 0.3–12.1) at RdGG and 0 (IQR 0–6.7) at OLVG.

## Discussion

This study shows that rTARUP is a safe and effective procedure. Recurrence rate was low at 2 years follow-up. A large majority of patients reported a better or much better quality of life after surgery.

Literature about rTARUP outcomes is scarce and mostly describes short-term follow-up. Follow-up studies of respectively 30 days and 6 weeks were reported by Ferraro et al. (81 patients) and by Baur et al. (30 patients) with no recurrences reported in either study [22, 23]. In the current study, a recurrence rate of 2.4% is found at a median follow-up of 24 months. Because we scheduled in-office visits at 2-year follow up on indication, it is possible that we underestimate the true rate of recurrence in our study, asymptomatic or subclinical recurrences may not have been detected. Our rate is comparable with other long term follow-up studies, describing recurrence rates varying between 1.48%–3.96% after 12–36 months of follow-up [8, 24, 25]. The longest series currently described follows 162 patients for 4.5 years with a recurrence rate of 3.7% [9]. Their method was of follow up was comparable to ours and consisted of telephonic follow up with additional in-office appointments on indication. Because they report on a follow up time that is twice ours, it is reasonable to assume our recurrence rate will also increase with time. It is important to note that two of our recurrences occurred cranially from the mesh where the rectus diastasis was not completely approximated. Possibly they were missed small epigastric hernias that progressed to clinical hernias. Diastasis recti recurrence occurred in two patients, who both had a BMI above 30.

Comparable recurrence rates are seen for rTARUP compared to open Rives-Stoppa. In a large meta-analysis by Den Hartog et al., consisting of 93 studies representing 12,440 patients, the recurrence rate after retro-rectus repair according to Rives-Stoppa was 3.2% at 12 months and 4.1% at 24 months. Den Hartog et al. also included studies in which recurrent hernias were treated, reporting a recurrence rate of 3.7% at 12 months for this subgroup [3]. For our study population, the recurrence rate in this subgroup was 5.4% at 24 months follow up.

Because rTARUP can create damage or weakness to the lateral border of the rectus sheath, some surgeons prefer eTEP to avoid a lateral incision. rTARUP/rTARM was decribed earlier than eTEP and eTEP can be seen as a logical next step after rTARUP in the development of robotic AWS. A possible disadvantage of eTEP is that it is even more technically challenging and that intra-peritoneal adhesions may be missed. We believe both techniques deserve a place in minimally invasive ventral hernia surgery. In a systematic review and meta-analysis by Brucchi et al., rTARUP was compared with Robot-assisted Enhanced-view Totally Extraperitoneal (r-eTEP). Three studies, representing 308 patients (r-eTEP 176, r-TARUP 132) were included for analysis [26]. There were no recurrences in either group reported at 35 months of follow up in the study by Olivier et al. [27]. The two other included studies also reported zero recurrences, at a maximum of 90 days follow-up [28, 29]. In the systematic review by Brucchi et al., comparing r-eTEP to rTARUP, no significant differences were found regarding major complications, recurrence or 30-day readmission. Minor complications occurred significantly more often in the rTARUP-group with a risk difference −0.14 for r-eTEP. However, because rTARUP was performed earlier in the learning curve and extensive adhesiolysis was required in 43.9% of patients, the authors suggest this perceived risk difference might be study-specific rather than an inherent advantage of the r-eTEP technique [26]. Brucchi et al. concludes that current evidence remains insufficient to favor one approach over the other, and more data is required to guide surgical decision making.

In the current study, we report a 30-day complication rate of 7.2%, including three (2.4%) major complications requiring a re-intervention. Complication rates were again highest in the recurrent hernia subgroup, with a rate of 16.2%. Lack of data from other studies makes it difficult to put these results in perspective. However, the results underline the complexity of treatment of this specific subgroup. To the best of our knowledge, no other studies regarding rTARUP outcomes have included recurrent hernias. In the primary hernia group complication rates were low (1.6%). These results are more favourable compared to other studies. Garza et al. reported surgical site complications requiring procedural intervention in 5% of patients, in which one hematoma was evacuated from the retromuscular space, and one seroma was drained. Other complications consisted of subcutaneous seromas/hematomas and a wound infection [8]. Muysoms et al. described patients (4.9%) needing surgical re-intervention due to one retromuscular hematoma and one superficial skin infection [7]. The study group published follow up data in an abstract-only paper, in which they report six (3%) readmissions within 30 days [24]. Baur et al. report a minor complication rate of 30%, and 6.7% of patients had major complications [23]. Because diastasis recti was present in 95% of our patient population, it is interesting to compare our data to the results of Cuccurullo et al. who studied 45 patients with ventral hernia and concomitant rectus diastasis. Their study population consisted of three-quarters primary hernia and one-quarter incisional hernia and were treated with rTARUP. No major complications were reported [25]. Ferraro et al. report a complication rate of 7.4%, comparable to our results [22].

This study shows that rTARUP can be safely performed in outpatient setting and can be performed in a timely manner with a median operative time of 134 min and a median surgical (skin to skin) time of 90 min. In the meta-analysis by Brucchi et al., operative times were 176 and 178 min for r-TEP and r-TARUP, respectively [26]. Operative times improved significantly over time. The difference in operative times between centers can be explained by differing levels of prior robotic experience between surgeons. Per protocol, RdGG patients were not operated as daycase. This was due to patients receiving intrathecal morphine and consequentially, a urinary catheter. At OLVG, the anesthesia protocol included general anesthesia without epidural or intrathecal pain administration and allowed for same day discharge.

We also address the importance of quality-of-life outcomes in our study, as opposed to only reporting on surgical outcomes. A methodical limitation of our study is that we only collected post-operative quality-of-life data and cannot compare the pre-operative quality of life. Of the entire study group, patients with incisional hernias had the best EuraHS-quality of life scores, with a total score of 6.66/90. Primary hernias were second with a median score of 9/90, and recurrent hernias were last with 10/90. Quality of life data after rTARUP is limited. Cuccurullo et al. described an improvement in mean total EuraHS-QoL score from 60/90 pre-operatively to 16/90 post-operatively. In the total domain scores, there was barely any pain or feeling of restriction in any of the subgroups. The cosmetic domain did least well with score of 4/20 or 5/20 in all subgroups. An important complaint was a postoperative central bulging of the midline closure. Therefore, it is important to properly council patients about the way the surgery affects the abdominal wall and that it does not treat any excess skin that has developed with the hernia. Further research is warranted into risk factors for cosmetic dissatisfaction and to determine the best treatment for ventral hernia patients with concomitant rectus diastasis, who are often young (post-partum) women in which cosmetic results may be of more importance.

Despite the favourable median pain scores, six patients (4.8%) reported experiencing chronic pain during follow up. Two patients were diagnosed with painful scar tissue. These patients stated the pain was manageable and were comforted with the knowledge it was not caused by recurrence. In four patients reporting chronic pain, prior investigations have ruled out conditions such as recurrence, mesh infection or other complications. The cause of pain in these cases remains unclear, which warrants further research into its origin. Known risk factors for the development of chronic pain after ventral hernia repair include acute pain before surgery, poorly managed pain in the direct post-operative period, and pain at 1-month post-surgery, as well as recurrent hernia, female sex and younger age [30, 31]. Clearly, it is important to ensure adequate pain management in the peri-operative period. Literature about chronic pain after minimally invasive retromuscular hernia repair is scarce. Krpata et al. studied the effect of medium-weight versus heavy-weight polypropylene mesh after open ventral hernia repair in a randomized controlled trial of 350 patients. At 30 days and at 1 year follow up, pain scores were identical in both groups, as well as other quality of life outcomes [32]. Post-herniorrhaphy chronic pain in inguinal hernia has been studied extensively, and estimations are that 8%–16% of annually performed groin hernia repair result in chronic pain that impairs daily lives for 6 months postoperatively [33]. Laparoscopic techniques seem to cause less pain than open techniques, as well as non-mesh-based techniques in dedicated centers. When heavyweight meshes were compared with lightweight meshes in laparoscopic groin hernia repair, results were comparable [33]. In groin hernia repair, chronic pain can be multifactorial, and it is often hard to make a definite diagnosis of the cause of the pain. Perioperative injury to nerves could lead to pain, as well as entrapment of nerves in a meshoma or in shrinkage of a mesh. Another way for mesh to cause pain could be by the inflammatory response to the mesh [33]. It is likely those same mechanisms are contributing to pain after minimally invasive ventral hernia repair, but more research is needed to determine contributors to chronic pain in this specific patient group.

This study has its limitations. Firstly, a retrospective analysis of consecutively collected series was performed, which brings risk in terms of bias. Secondly, we cannot compare the quality of life pre- and post-operatively due to the missing pre-operative quality-of-life data. During prospective follow up, we could not reach all patients. Our response rate of 77.6% is acceptable, but it could potentially over- or underestimate our quality-of-life outcomes. Lastly, all surgeries were performed by experienced hernia surgeons, which limits the applicability of our results to surgeons with similar levels of experience.

Strengths of this study were the long follow up period and the multicentered character of the study, broadening generalizability. Because the study was conducted at top-clinical ventral hernia repair centers, we were willing to treat and include patients undergoing repair for recurrent hernias or patients with extensive abdominal history. This shows the applicability of this procedure in a wide group of hernia patients. We also gathered quality of life data during follow up, which adds to the patient perspective of this new technique and provides us with ways on how to further improve the operation itself and the post-operative period. Both surgeons have broadened techniques for this category of patients to rTAPP and eTEP in certain indications although rTARUP is deemed the default operation, especially in the beginning of learning curves.

Economical costs and benefits were outside the scope of this study but are important to address. The robotic approach could be more economically favourable when considering the decreased length of hospital stay, the low complication and recurrence rates, and that patients are generally able to return to work sooner as compared to other, open techniques. More research is needed to determine costs and benefits and to be able to economically justify robotic surgery in abdominal wall repairs and [22, 35].

## Conclusion

This study about rTARUP/rTARM shows encouraging results regarding safety and efficacy. With experience, rTARUP can be carried out in a timely manner and performed in day care. Because potential short hospital stay and operative times, rTARUP could eventually be more cost-effective than open approaches in the treatment of ventral hernia. The study also demonstrates the safe and efficacious use of rTARUP in patients with recurrent hernias. Although perioperative complications occur more often in this group, recurrence rates were low, indicating that rTARUP might offer a solution for this complex patient population. More research is warranted into the development of long-term complications such as chronic pain after minimally invasive ventral hernia repair and into economical costs and benefits. Another opportunity for research is to determine best treatment options for ventral hernia patients with concomitant rectus diastasis and risk factors for cosmetic dissatisfaction.

## Data Availability

The raw data supporting the conclusions of this article will be made available by the authors, without undue reservation.

## References

[1] Ezeme C Mackenzie P Newton RC . Ventral Hernias: Understanding the Pathogenesis, Prevention and Repair. Surgery (Oxford) (2024) 42(1):22–32. 10.1016/j.mpsur.2023.11.007

[2] Gillies M Anthony L Al-Roubaie A Rockliff A Phong J . Trends in Incisional and Ventral Hernia Repair: A Population Analysis from 2001 to 2021. Cureus (2023) 15(3):e35744. 10.7759/cureus.35744 36879583 PMC9984720

[3] Hartog F Sneiders D Darwish EF Yurtkap Y Menon AG Muysoms FE Favorable Outcomes After Retro-Rectus (Rives-Stoppa) Mesh Repair as Treatment for Noncomplex Ventral Abdominal Wall Hernia, a Systematic Review and Meta-Analysis. Ann Surg (2022) 276(1):55–65. 10.1097/SLA.0000000000005422 35185120

[4] Schroeder AD Debus ES Schroeder M Reinpold WM . Laparoscopic Transperitoneal Sublay Mesh Repair: A New Technique for the Cure of Ventral and Incisional Hernias. Surg Endosc (2013) 27(2):648–54. 10.1007/s00464-012-2508-9 22955899

[5] Mitura K . New Techniques in Ventral Hernia Surgery - an Evolution of Minimally-Invasivehernia Repairs. Pol Przegl Chir (2020) 92(4):38–46. 10.5604/01.3001.0014.1898 32908011

[6] Mitura K . Different Approach to Laparoscopic IPOM Ventral Hernia Surgery -What Has the Last Decade Taught Us? Polski Przeglad Chirurgiczny (2016) 88:54–61. 10.1515/pjs-2016-0028 27096776

[7] Muysoms F Van Cleven S Pletinckx P Ballecer C Ramaswamy A . Robotic Transabdominal Retromuscular Umbilical Prosthetic Hernia Repair (TARUP): Observational Study on the Operative Time During the Learning Curve. Hernia (2018) 22(6):1101–11. 10.1007/s10029-018-1825-x 30244344

[8] Garza A Amaya-Romero C Arevalo G . Outcomes of Robotic Transabdominal Retromuscular Repair: 3-Year Follow-Up. J Abdom Wall Surg (2024) 3:12907. 10.3389/jaws.2024.12907 38966856 PMC11222322

[9] Vierstraete M De Troyer A Pletinckx P Hermie E Muysoms F . Lateral Single-Dock Robot-Assisted Retro-Rectus Ventral Hernia Repair (rTARUP/rTARM): Observational Study on Long-Term Follow-Up. J Robotic Surg (2025) 19(1):84. 10.1007/s11701-025-02243-2 40014163 PMC11868346

[10] Henriksen NA Montgomery A Kaufmann R Berrevoet F East B Fischer J Guidelines for Treatment of Umbilical and Epigastric Hernias From the European Hernia Society and Americas Hernia Society. Br J Surg (2020) 107(3):171–90. 10.1002/bjs.11489 31916607

[11] Reinpold W Köckerling F Bittner R Conze J Fortelny R Koch A Classification of Rectus Diastasis—A Proposal by the German Hernia Society (DHG) and the International Endohernia Society (IEHS). Front Surg (2019) 6:1. 10.3389/fsurg.2019.00001 30746364 PMC6360174

[12] Muysoms FE Miserez M Berrevoet F Campanelli G Champault GG Chelala E Classification of Primary and Incisional Abdominal Wall Hernias. Hernia (2009) 13(4):407–14. 10.1007/s10029-009-0518-x 19495920 PMC2719726

[13] Petro CC O'Rourke CP Posielski NM Criss CN Raigani S Prabhu AS Designing a Ventral Hernia Staging System. Hernia (2016) 20(1):111–7. 10.1007/s10029-015-1418-x 26342924

[14] Clavien PA Barkun J de Oliveira ML Vauthey JN Dindo D Schulick RD The Clavien-Dindo Classification of Surgical Complications: Five-Year Experience. Ann Surg (2009) 250(2):187–96. 10.1097/SLA.0b013e3181b13ca2 19638912

[15] Muysoms F Campanelli G Champault GG DeBeaux AC Dietz UA Jeekel J Eurahs: The Development of an International Online Platform for Registration and Outcome Measurement of Ventral Abdominal Wall Hernia Repair. Hernia (2012) 16(3):239–50. 10.1007/s10029-012-0912-7 22527930 PMC3360853

[16] Slater NJ Montgomery A Berrevoet F Carbonell AM Chang A Franklin M Criteria for Definition of a Complex Abdominal Wall Hernia. Hernia (2014) 18(1):7–17. 10.1007/s10029-013-1168-6 24150721

[17] Flum DR Horvath K Koepsell T . Have Outcomes of Incisional Hernia Repair Improved With Time? A Population-Based Analysis. Ann Surg (2003) 237(1):129–35. 10.1097/00000658-200301000-00018 12496540 PMC1513979

[18] Korenkov M Paul A Sauerland S Neugebauer E Arndt M Chevrel JP Classification and Surgical Treatment of Incisional Hernia. Results of an Experts' Meeting. Langenbecks Arch Surg (2001) 386(1):65–73. 10.1007/s004230000182 11405092

[19] Chowbey PK Khullar R Mehrotra M Sharma A Soni V Baijal M . Sir Ganga Ram Hospital Classification of Groin and Ventral Abdominal Wall Hernias. J Minim Access Surg (2006) 2(3):106–9. 10.4103/0972-9941.27720 21187888 PMC2999767

[20] Dietz UA Hamelmann W Winkler MS Debus ES Malafaia O Czeczko NG An Alternative Classification of Incisional Hernias Enlisting Morphology, Body Type and Risk Factors in the Assessment of Prognosis and Tailoring of Surgical Technique. J Plast Reconstr Aesthet Surg (2007) 60(4):383–8. 10.1016/j.bjps.2006.10.010 17349593

[21] Rudmik LR Schieman C Dixon E Debru E . Laparoscopic Incisional Hernia Repair: A Review of the Literature. Hernia (2006) 10(2):110–9. 10.1007/s10029-006-0066-6 16453075

[22] Ferraro L Formisano G Salaj A Giuratrabocchetta S Toti F Felicioni L Preliminary Robotic Abdominal Wall Reconstruction Experience: Single-Centre Outcomes of the First 150 Cases. Langenbeck's Arch Surg (2023) 408(1):276. 10.1007/s00423-023-03004-1 37450034

[23] Baur J Ramser M Keller N Muysoms F Dörfer J Wiegering A Robotic Hernia Repair II. English Version: Robotic Primary Ventral and Incisional Hernia Repair (rv-TAPP and r-Rives or r-TARUP). Video Report and Results of a Series of 118 Patients. Chirurg (2021) 92(Suppl. 1):15–26. 10.1007/s00104-021-01479-6 34928426 PMC8695526

[24] Muysoms F Dewulf M Nachtergaele F Pletinckx P . P140 Observational Study with One Year Follow-Up on Robotic Assisted Retro-Rectus Ventral Hernia Repair (Rtarup) with A Self-Fixating Mesh. Br J Surg (2021) 108(Suppl. ment_8):znab395.132. 10.1093/bjs/znab395.132

[25] Cuccurullo D Guerriero L Mazzoni G Sagnelli C Tartaglia E . Robotic Transabdominal Retromuscular Rectus Diastasis (r-TARRD) Repair: A New Approach. Hernia (2022) 26(6):1501–9. 10.1007/s10029-021-02547-w 34982294

[26] Brucchi F De Troyer A Sassun R Dionigi G Muysoms F . Comparison of Robot-Assisted Enhanced-View Totally Extraperitoneal (Etep) and Transabdominal Retromuscular (TARM Aka TARUP) Ventral Hernia Mesh Repair: A Systematic Review and Meta-Analysis. J Abdom Wall Surg (2025) 4:14723–2025. 10.3389/jaws.2025.14723 40689023 PMC12270933

[27] Olivier F Abasbassi M Geers J . Robotic Retromuscular Abdominal Wall Repair Using an Inverted TEP (Itep) Approach: Surgical Technique and Short-Term Outcomes. Langenbeck's Arch Surg (2022) 407(5):2177–86. 10.1007/s00423-022-02561-1 35612661

[28] Pacheco TBS Hakmi H Halpern R Sohail AH Akerman M Weinman K A Comparison of Robotic Enhanced-View Totally Extraperitoneal Approach Versus Trans-Abdominal Retro-Muscular Approach for Midline Ventral Hernias. Hernia (2024) 28(5):1719–26. 10.1007/s10029-024-03042-8 38668808

[29] Kudsi OY Chang K Bou-Ayash N Gokcal F . Transabdominal (TA) Versus Totally Extraperitoneal (TEP) Robotic Retromuscular Ventral Hernia Repair: A Propensity Score Matching Analysis. Surg Endosc (2020) 34(8):3550–9. 10.1007/s00464-020-07574-9 32500458

[30] Cox TC Huntington CR Blair LJ Prasad T Lincourt AE Heniford BT Predictive Modeling for Chronic Pain After Ventral Hernia Repair. The Am J Surg (2016) 212(3):501–10. 10.1016/j.amjsurg.2016.02.021 27443426

[31] Salgaonkar H Wijerathne S Lomanto D . Managing Complications in Laparoscopic Ventral Hernia. Ann Laparosc Endoscopic Surg (2019) 4:11. 10.21037/ales.2019.01.04

[32] Krpata DM Petro CC Prabhu AS Tastaldi L Zolin S Fafaj A Effect of Hernia Mesh Weights on Postoperative Patient-Related and Clinical Outcomes After Open Ventral Hernia Repair: A Randomized Clinical Trial. JAMA Surg (2021) 156(12):1085–92. 10.1001/jamasurg.2021.4309 34524395 PMC8444061

[33] Andresen K Rosenberg J . Management of Chronic Pain After Hernia Repair. J Pain Res (2018) 11:675–81. 10.2147/JPR.S127820 29670394 PMC5896652

[34] Henriksen NA Marckmann M Christoffersen MW Jensen KK . Cost Analysis of Open Versus Robot-Assisted Ventral Hernia Repair – A Retrospective Cohort Study. Hernia (2024) 28(5):1823–9. 10.1007/s10029-024-03089-7 38922513 PMC11449943

[35] Feneley RC Hopley IB Wells PN . Urinary Catheters: History, Current Status, Adverse Events and Research Agenda. J Med Eng Technol (2015) 39(8):459–70. 10.3109/03091902.2015.1085600 26383168 PMC4673556

