# Supplementary material for: Outcomes of Robot Assisted Trans Abdominal Retromuscular Umbilical Prosthesis (rTARUP): A Dutch Multicenter Study
Source: J Abdom Wall Surg. 2025 Dec 22;4:15479. doi: 10.3389/jaws.2025.15479 (PMC12765712; doi:10.3389/jaws.2025.15479)
Supplement: Supplementary file 1 [file Supplementaryfile1.docx]

**Appendices**

*Appendix 1. Hernia width classification according to the European Hernia Society*

|  | W1 (small) | W2 (medium) | W3 (large) |
| --- | --- | --- | --- |
| Primary hernia | <2 cm | 2-4 cm | >4 cm |
| Incisional hernia | <4 cm | 4-10 cm | >10 cm |

*Appendix 2. HPW-classification for ventral hernias*

|  | H1 | H2 | H3 |
| --- | --- | --- | --- |
| P0 | Stage 1 | Stage 2 | Stage 3 |
| P1 | Stage 2 | Stage 3 | Stage 4 |
| W1 | Stage 3 | Stage 3 | Stage 4 |

H: hernia defect H1:<10; H2: 10-20; H3:>20 cm

P: patient factors: morbid obesity, diabetes, smoking, immunosuppressant use (none: P0, any factor or more present: P1)

W1: contaminated wound present

*Appendix 3. EuraHS Quality of Life scale*

1. Pain at the site of the hernia (0 = no pain, 10 = worst pain imaginable)

| Pain in rest (lying down) | 0 | 1 | 2 | 3 | 4 | 5 | 6 | 7 | 8 | 9 | 10 |
| --- | --- | --- | --- | --- | --- | --- | --- | --- | --- | --- | --- |
| Pain during activities (walking, biking, sports) | 0 | 1 | 2 | 3 | 4 | 5 | 6 | 7 | 8 | 9 | 10 |
| Pain felt during the last week | 0 | 1 | 2 | 3 | 4 | 5 | 6 | 7 | 8 | 9 | 10 |

1. Restriction of activities because of pain or discomfort at the site of the hernia (0 = no restriction, 10 = completely restricted, X = if you do not perform this activity)

| Restriction from daily activities (inside the house) | 0 | 1 | 2 | 3 | 4 | 5 | 6 | 7 | 8 | 9 | 10 | X |
| --- | --- | --- | --- | --- | --- | --- | --- | --- | --- | --- | --- | --- |
| Restriction outside the house (walking, biking, driving) | 0 | 1 | 2 | 3 | 4 | 5 | 6 | 7 | 8 | 9 | 10 | X |
| Restriction during sports | 0 | 1 | 2 | 3 | 4 | 5 | 6 | 7 | 8 | 9 | 10 | X |
| Restriction during heavy labour | 0 | 1 | 2 | 3 | 4 | 5 | 6 | 7 | 8 | 9 | 10 | X |

1. Esthetical discomfort (0 = very beautiful, 10 = extremely ugly)

| Shape of your abdomen | 0 | 1 | 2 | 3 | 4 | 5 | 6 | 7 | 8 | 9 | 10 |
| --- | --- | --- | --- | --- | --- | --- | --- | --- | --- | --- | --- |
| Site of the hernia | 0 | 1 | 2 | 3 | 4 | 5 | 6 | 7 | 8 | 9 | 10 |

*Appendix 4: Quality of life comparison between centers*

| **Quality of life outcomes** | **OLVG** | **RDGG** | **Total** | **P-value** |
| --- | --- | --- | --- | --- |
| QoL total score (/90)* (IQR) | 7 (0-20) | 12 (4-26) | 10 (2.25-24.3) | 0.089^A^ |
| QoL domain pain (/30)* (IQR) | 0 (0-2) | 2 (0-9) | 0 (0-4.25) | 0.003^A^ |
| QoL domain restrictions (/40)* (IQR) | 0 (0-6.67) | 4 (0.25-12.08) | 0 (0-8) | 0.003^A^ |
| QoL domain cosmesis (/20)* (IQR) | 5 (0-10) | 4 (2-10) | 4 (0-10) | 0.578^A^ |
| Patient-reported pain (%) | 3 (4.3) | 3 (5.4) | 6 (4.8) | 1.000^B^ |
| Worse or better off (%) |  |  |  | 0.624^C^ |
| Much worse | 2 (3.6) | 0 | 2 (2.5) |  |
| Worse | 1 (1.8) | 0 | 1 (1.3) |  |
| The same | 6 (10.9) | 4 (16) | 10 (12.5) |  |
| Better | 19 (34.5) | 12 (48.0) | 31 (38.8) |  |
| Much better | 27 (49.1) | 9 (36.0) | 36 (45.0) |  |

*not-normal distribution according to Shapiro-Wilk, ^A^Mann-Whitney Test, ^B^Fisher’s Exact Test (2-sided), ^C^Fisher-Freeman-Halton Exact Test (2-sided).

QoL, Quality of Life score according to the EuraHS-Quality of Life questionnaire; IQR, inter-quartile range.
